# Insights into microbial communities mediating the bioremediation of hydrocarbon-contaminated soil from an Alpine former military site

**DOI:** 10.1007/s00253-018-8932-6

**Published:** 2018-03-29

**Authors:** José A. Siles, Rosa Margesin

**Affiliations:** Institute of Microbiology, University of Innsbruck, Technikerstrasse 25, A-6020 Innsbruck, Austria

**Keywords:** Petroleum hydrocarbons, Hydrocarbon-contaminated soils, Bioremediation, Natural attenuation, Biostimulation, Soil bacteria, archaea and fungi

## Abstract

The study of microbial communities involved in soil bioremediation is important to identify the specific microbial characteristics that determine improved decontamination rates. Here, we characterized bacterial, archaeal, and fungal communities in terms of (i) abundance (using quantitative PCR) and (ii) taxonomic diversity and structure (using Illumina amplicon sequencing) during the bioremediation of long-term hydrocarbon-contaminated soil from an Alpine former military site during 15 weeks comparing biostimulation (inorganic NPK fertilization) vs. natural attenuation and considering the effect of temperature (10 vs. 20 °C). Although a considerable amount of total petroleum hydrocarbon (TPH) loss could be attributed to natural attenuation, significantly higher TPH removal rates were obtained with NPK fertilization and at increased temperature, which were related to the stimulation of the activities of indigenous soil microorganisms. Changing structures of bacterial and fungal communities significantly explained shifts in TPH contents in both natural attenuation and biostimulation treatments at 10 and 20 °C. However, archaeal communities, in general, and changing abundances and diversities in bacterial and fungal communities did not play a decisive role on the effectiveness of soil bioremediation. *Gammaproteobacteria* and *Bacteroidia* classes, within bacterial community, and undescribed/novel groups, within fungal community, proved to be actively involved in TPH removal in natural attenuation and biostimulation at both temperatures.

**Electronic supplementary material:**

The online version of this article (10.1007/s00253-018-8932-6) contains supplementary material, which is available to authorized users.

## Introduction

Many places around the world are impacted by petroleum hydrocarbon contamination as a consequence of anthropogenic activities such as industrial and municipal runoffs, effluent release, and offshore and onshore petroleum industry as well as accidental spills, which has become a major environmental concern (Brassington et al. 2007; Gkorezis et al. 2016). The United States Environmental Protection Agency has classified specific petroleum hydrocarbons such as some polycyclic aromatic hydrocarbons (PAHs) as priority environmental pollutants because of their negative impact on quality services of ecosystems, animal life, and human health (Marco-Urrea et al. 2015; Safdari et al. 2018).

The development of effective strategies for the decontamination of petroleum hydrocarbon-contaminated environments is thus needed. Physical and chemical strategies, such as chemical inactivation, combusting, photolysis, soil vapor extraction, and soil washing, have been used as decontamination strategies for petroleum-polluted soils (Varjani 2017; Xu et al. 2013). However, these methods are not economically viable and can involve significant site disturbances (Shahi et al. 2016a). As an alternative, bioremediation, taking advantage of the capacity of a wide range of microorganisms to use hydrocarbons as the sole source of carbon and energy (biodegradation), has shown to be a cost-effective and ecologically acceptable clean-up approach to treat hydrocarbon-contaminated soils (Varjani 2017). Bioremediation treatments have been extensively applied for the decontamination of soils from an ample range of environments using laboratory or in situ approaches (Azubuike et al. 2016; Margesin 2007; Varjani 2017). Treatments based on biostimulation (addition of appropriate nutrients to stimulate the degradation capacity of the indigenous soil microbial community) or bioaugmentation (soil inoculation with hydrocarbon-degrading microorganisms) attempt to optimize limiting environmental conditions. Biostimulation has shown to be the most effective approach for the bioremediation of historically contaminated soils with petroleum hydrocarbons, where microbial communities are adapted to the existing physicochemical and environmental conditions as well as to the prevailing pollutants (Margesin and Schinner 2005; Megharaj et al. 2011; Tyagi et al. 2011). The lack of a balanced C:N:P (carbon:nitrogen:phosphorus) ratio constrains most bioremediation processes that involve indigenous soil microorganisms (Gkorezis et al. 2016; Safdari et al. 2018). Among other limiting abiotic factors (e.g., soil pH, oxygen content, soil structure, age, concentration, and composition of contamination), temperature plays a major role.

During the bioremediation of hydrocarbon-contaminated soils, the characterization of the involved microorganisms is crucial since the final success of the biodegradation processes greatly depends on soil microbial features in terms of activity, abundance, diversity, and community structure. Data obtained from this kind of studies are useful to optimize the existing bioremediation strategies and to develop new ones (Pal et al. 2017; Stenuit et al. 2008). In this regard, the study of the diversity and structure of the degradative soil microbial communities has shown to be especially valuable (Bao et al. 2017; Wu et al. 2017). Previous works have applied high-throughput sequencing techniques to study the dynamics of key bacterial (Shahi et al. 2016b; Singleton et al. 2013; Wu et al. 2017) and fungal groups (Covino et al. 2016) during biostimulation. Although soil hydrocarbon degraders may include groups of *Bacteria*, *Archaea*, *Fungi*, and algae (Militon et al. 2010), the number of surveys simultaneously studying the dynamics of microbial communities of all three microbial domains involved in bioremediation is still scarce.

In an earlier laboratory feasibility study on the bioremediation of a long-term hydrocarbon-contaminated soil from an Alpine former military site over a period of 30 weeks, we demonstrated the significant impact of temperature on hydrocarbon loss, microbial activity, and community structure (phospholipid fatty acids, PLFAs) (Mair et al. 2013). Biostimulation by the addition of nutrients had a significantly stimulating effect on the biodegradation activity of the indigenous soil microorganisms; however, a considerable amount of hydrocarbon loss could be attributed to natural attenuation. PLFA-based analysis of soil microbial communities demonstrated the involvement of Gram-negative and Gram-positive bacteria and fungi in bioremediation and the significant interaction of temperature and nutrient addition. Shifts in microbial community composition during bioremediation included the significant increase of soil fungi at 10 °C and of Gram-negative soil bacteria at 20 °C (Mair et al. 2013). However, a more detailed analysis of the microbial community involved in the various biostimulation treatments was not possible with the applied approach. A study on the culturable heterotrophic bacterial community in the soil at the beginning of bioremediation (t0) revealed the dominance of *Proteobacteria* (mainly *Alphaproteobacteria*) over *Firmicutes*, *Actinobacteria*, and *Bacteroidetes* (Zhang and Margesin 2014). It was the objective of the present study to complement our previous works by deeply characterizing soil microbial communities, including bacterial and archaeal domains as well as fungal kingdom, in terms of (i) abundance (using quantitative PCR) and (ii) diversity as well as taxonomic composition (using Illumina amplicon sequencing). Since it was not possible to analyze all samples collected from all treatments over 30 weeks, we compared natural attenuation with inorganic NPK fertilization, taking into consideration temperature (10 and 20 °C), at the beginning and in the middle (15 weeks) of the bioremediation study, when already a substantial loss of total petroleum hydrocarbons (TPH) had occurred. We hypothesized that higher bacterial, archaeal, and fungal abundances in concomitance with lower diversities and changing community structures (due to the selective proliferation of hydrocarbon-degrading groups) would characterize increased rates of hydrocarbons removal.

## Materials and methods

### Bioremediation study

The petroleum hydrocarbon-contaminated soil investigated in the present study was collected from an Alpine former military site in Welsberg-Taisten (46° 45′ 10.57″ N; 12° 06′ 47.39″ E), South Tyrol, Italy. Annual air temperature in the sampling area ranges from – 15 to +25 °C, and the mean annual temperature is 7–9 °C. At the time of sampling, the mean soil temperature was 10–18 °C. A detailed description of the site has previously been reported (Mair et al. 2013). The soil was a mixture of gravel, sand, and clay and was characterized by a pH of 6.1–6.2 and low contents of N and P (< 3 mg kg^−1^ soil) and carbonate (3–4%). The concentration of TPH (which primarily consist of alkanes, aromatics, and nitrogen-, sulfur-, and oxygen-containing compounds (Dong et al. 2015)) in the range of C_10_ to C_40_ was 6220 mg kg^−1^ dry mass soil (Mair et al. 2013). The hydrocarbon contamination could be attributed to diesel oil; the presence of heavy oil was not detected.

The feasibility study described by Mair et al. (2013) included the determination of the effects of temperature (10 and 20 °C) and of various biostimulation treatments (inorganic NPK fertilization and the two commercial products Inipol EAP22 and Terramend) vs. natural attenuation on the loss of TPH, microbial activity (soil respiration), and community composition (PLFA-based) over a period of 30 weeks. For the characterization of soil microbial communities in the present study, the following treatments were selected: (i) natural attenuation: soil without fertilization, incubated for 15 weeks at 10 °C (UNF10) and at 20 °C (UNF20) and (ii) biostimulation: soil fertilized with an agricultural inorganic NPK fertilizer (containing 9.5% NH_3_-N, 5.5% NO_3_-N, 6.6% P_2_O_5_-P, and 12.2% K_2_O-K) at a C:N ratio of 20:1 according to the initial hydrocarbon concentration (as previously justified (Mair et al. 2013; Margesin et al. 2007)), incubated for 15 weeks at 10 °C (NPK10) and at 20 °C (NPK20). The N concentration in soil solution was 2230 mg N kg^−1^ soil H_2_0, which is in agreement with those levels regarded as optimal by Walworth et al. (2007). Untreated soil at the beginning of the feasibility study was used as control (t0). At t0 and after incubation time, three samples were collected from each treatment under sterile conditions, analyzed (Mair et al. 2013), and stored at −80 °C. These samples were thawed for the present study and used for DNA extraction.

The selection of treatments for the present study was based on the comparison of soil microbial communities at the beginning (t0) and in the middle of the experiment (after 15 weeks), where a considerable part of TPH contents had already been removed and data for microbial activity (soil respiration) and PLFA-derived characterization of microbial communities were available. With regard to biostimulation treatments, NPK fertilization was selected because of its highest positive impact on TPH removal after 15 weeks at 20 °C and on soil respiration at both test temperatures.

### DNA extraction

Total DNA from each sample (5 treatments × 3 replicates = 15 samples) was extracted in triplicate from 250 mg of soil fresh mass using Power Soil™ DNA Isolation Kit (MO BIO Laboratories Inc., Solana Beach, USA) following the manufacturer’s instructions. Triplicate DNA extracts from each sample were then pooled, and DNAs were purified using DNA Clean & Concentrator Kit (Zymo Research, Irvine, USA) according to manufacturer’s instructions. The quality of DNAs was spectrophotometrically checked by NanoDrop (Thermo Fisher Scientific Inc., Bremen, Germany) based on the absorbance ratios A_260_/A_280_ and A_260_/A_230_. Subsequently, DNAs were quantified using QuantiFluor™ dsDNA System (Promega, Madison, USA) and DNA concentration for each sample was standardized to 1 ng μL^−1^.

### Quantitative PCR analyses

Quantitative PCR analysis was conducted to determine the abundances of bacterial and archaeal 16S rRNA, fungal 18S rRNA, and *gtlA* (bacterial citrate synthase gene, as functional marker of aerobic bacterial respiration) genes using the pairs of primers Eub338/Eub518 for bacteria (Fierer et al. 2005), Arch-967F/Arch-1060R for archaea (Cadillo-Quiroz et al. 2006), FR1/FF390 for fungi (Chemidlin Prevost-Boure et al. 2011), and CS680F/CS904R for *gtlA* gene (Castro et al. 2012). The domain or kingdom specificity of each pair of primers was confirmed using the RDP (ribosomal database project) probe match tool (Cole et al. 2014). Each DNA sample was analyzed in duplicate on a Corbett Life Science Rotor-Gene 6000 system (Qiagen, Valencia, USA). Single qPCR reactions were carried out containing 10 μL of iQ™ SYBR® Green Supermix (Bio-Rad, Hercules, USA), 0.4 μL of each primer (10 μM) (Eurofins MWG, Ebersberg, Germany), 0.4 μL of bovine serum albumin (10 mg mL^−1^) (New England Biolabs, Hitchin, UK), 2 μL of DNA (1 ng μL^−1^), and 6.8 μL of H_2_O, under the following thermal conditions: 95 °C for 10 min followed by 40 cycles of 95 °C for 20 s, 53 °C (bacteria)/59 °C (archaea)/50 °C (fungi)/61 °C (*gtlA* gene) for 20 s, and 72 °C for 30 s. In all the cases, after amplification reactions, melting curve and gel electrophoresis analyses were conducted to confirm that the amplified products had the appropriate size. Copy numbers for each gene were calculated using a regression equation for each assay relating the cycle threshold (Ct) value to the known number of copies in the serially diluted standards of a plasmid standard curve containing the appropriate target gene. Construction of plasmid standard curves was done as previously reported (Siles and Margesin 2016).

### Illumina amplicon sequencing

Prokaryotic communities were characterized in terms of taxonomic diversity and composition by amplifying the V4–V5 hypervariable region of 16S rRNA gene using the universal bacterial and archaeal primers new515F (5′-GTGYCAGCMGCCGCGGTAA-3′) and 909R (5′-CCCCGYCAATTCMTTTRAGT-3′) (Tamaki et al. 2011; Walters et al. 2015). Each DNA sample was amplified using the HotStarTaq *Plus* Master Mix Kit (Qiagen, Valencia, USA) and barcoded forward primers, under the thermal conditions previously described by Siles and Margesin (2017). The success of the amplifications was checked in a 2% agarose gel and PCR products were subsequently purified using Agencourt AMPure XP magnetic beads kit (Beckman Coulter, Inc., Pasadena, USA), quantified with the QuantiFluor™ dsDNA System and pooled in equal proportions. The pooled product was then used to prepare Illumina DNA library following TruSeq DNA library preparation protocol. Paired-end sequencing (2 × 300) was performed on the Illumina MiSeq sequencing platform (Illumina, San Diego, USA) at MR DNA (www.mrdnalab.com, Shallowater, TX, USA).

Fungal communities were analyzed by amplification of ITS (internal transcribed spacer) 1 region using the ITS1F (5′-CTTGGTCATTTAGAGGAAGTAA-3′) and ITS2 (5′-GCTGCGTTCTTCATCGATGC-3′) primers (Gardes and Bruns 1993; White et al. 1990) under the cycling conditions previously described (Siles and Margesin 2017). The processing of amplicons for paired-end sequencing (2 × 300) on Illumina MiSeq sequencing platform was as described for prokaryotic community.

The raw prokaryotic and fungal sequences associated with this study were deposited in the GenBank SRA database under BioProject accession number PRJNA418398.

### Processing of Illumina sequencing data

First, raw MiSeq paired-end reads from 16 rRNA gene and ITS region were separately assembled and reoriented using MR DNA pipeline. Subsequently, reads were demultiplexed and formatted for processing using a Phython script (Siles and Margesin 2016). Sequences were then processed using USEARCH pipeline and UPARSE-OTU algorithm (Edgar 2013). Briefly, reads were separately quality-filtered allowing a maximum *e* value of 1.0 for both sets of libraries, trimmed (to 300-bp (base pair) and 250-bp for prokaryotic and fungal libraries, respectively), dereplicated, and sorted by abundance (removing singleton sequences), prior chimera detection and OTU (operational taxonomic unit) determination at 97% sequence identity. Finally, the original trimmed and high-quality sequences were mapped to OTUs at the 97% identity threshold obtaining one OTU table for prokaryotic community and another one for fungal community. The taxonomic affiliation of each OTU was obtained using RDP taxonomic classifier (Wang et al. 2007) against 16S rRNA training set 16 for 16s rRNA gene sequences and UNITE Fungal ITS trainset 07-04-2014 for ITS sequences with a 50% confidence threshold in both cases. Next, the prokaryotic OTU table was divided into two different OTU tables, one of them containing those OTUs classified as *Bacteria* and another one containing those classified as *Archaea*. All the OTUs from fungal OTU table were successfully classified as *Fungi* (kingdom level) and were thus retained. These three OTU tables were used for downstream analyses. However, in the case of *Archaea*, since the number of sequences obtained across the different libraries was highly heterogeneous, with some of them containing a very small number of reads, the data were used only to investigate shifts in taxonomic composition of the archaeal community after 15 weeks in the absence/presence of nutrients, at 10 °C and/or at 20 °C, in comparison to the soil archaeal community at t0. For bacterial and fungal OTU tables, the sequencing depth across libraries was normalized to 78,000 and 22,700, respectively. These two normalized OTU tables were used for downstream analyses, including the calculation of the relative abundance of the different taxonomic groups.

### Statistical and diversity analyses

One-way ANOVA (analysis of variance) was applied to determine whether fertilization and/or temperature after 15 weeks, compared to conditions at t0, had a significant effect (*p* ≤ 0.05) on (i) qPCR-based microbial abundances and potential aerobic bacterial respiration, (ii) bacterial and fungal diversity measurements, and (iii) relative abundance of the different bacterial, archaeal, and fungal taxonomic groups. When one-way ANOVA resulted in significant results, Tukey’s HSD (honest significance difference) post-hoc test was used for multiple comparisons of means at a 95% confidence interval. MANOVA (multifactor ANOVA) was applied to check the significance of the factors fertilization (presence and absence) and temperature on the abundance and diversity characteristics of microbial communities. Normality and heteroscedasticity of data were tested by the Shapiro-Wilk and Levene tests, respectively. In case that one of these conditions was not met, the values were transformed using the natural logarithm.

The bacterial and fungal communities were characterized in terms of diversity by calculating richness (number of OTUs), the Shannon index, richness estimator index ACE (abundance-based coverage estimation), and Smith-Wilson evenness by using Mothur v.1.39.5 (Schloss et al. 2009). NMDS (non-metric multidimensional scaling) was chosen as ordination method to visualize patterns in OTU-based bacterial and fungal community structures. The significance of the variations in the structure of bacterial and fungal communities was tested using PERMANOVA (permutational analysis of variance), ANOSIM (analysis of similarity), and MRPP (multiresponse permutation procedure) with Bray-Curtis similarities and 9999 permutations. All the analyses were done using the package “vegan” in R (Oksanen et al. 2013). SIMPER (similarity percentage) analysis was applied to assess the average dissimilarity among the studied treatments and to identify the bacterial and fungal OTUs primarily responsible for the observed dissimilarities using Bray-Curtis similarities with the software PAST ver. 3.07 (Hammer et al. 2008). The Mantel test was used to study the relationship between the TPH content and OTU-based structure of bacterial and fungal communities using Bray-Curtis similarities and 9999 permutations with the package “vegan” in R. Pearson correlations (using log-transformed data when they did not meet the normality assumptions) were used to find significant links between TPH contents and the properties of microbial communities in the studied treatments.

## Results

### Total petroleum-derived hydrocarbon content

After 15 weeks, the relative TPH degradation (as percentage of the initial TPH content) at 10 °C was not significantly influenced by the presence/absence of NPK nutrients (UNF10 47.5%; NPK10 46.5%). However, TPH loss at 20 °C was much higher in the NPK-fertilized soil (NPK20 74.7%) than after natural attenuation (UNF20 55.2%) (Mair et al. 2013). Soil TPH decontamination was thus significantly favored by biostimulation and increased (20 vs. 10 °C) temperature.

### Microbial abundance and potential aerobic bacterial respiration

NPK fertilization (biostimulation) had resulted in a significant increase in qPCR-based bacterial abundances with respect to natural attenuation and the control soil at initial conditions after 15 weeks (Table 1). Yet, the opposite was noted for *Archaea*. For both bacterial and archaeal communities, a higher temperature (20 vs. 10 °C) did not result in significantly increased abundances (the factor temperature and the interaction fertilization×temperature were thus not significant). Changes in fungal communities in terms of abundance responded to a significant effect of the interaction nutrients (absence/presence) and temperature (*F* = 29.16; *p* < 0.0001); an increase in temperature resulted in higher fungal abundances in the absence of fertilization, but not in the presence of NPK, where fungal abundances even decreased compared to t0. Results for ratios A/B and F/B went in parallel with those obtained for archaeal and fungal abundances. The copy number of *gtlA* gene, as a measurement of potential aerobic bacterial respiration, had significantly increased after 15 weeks in NPK-fertilized soil and at 20 °C (significant effect of the factors fertilization and temperature as well as their interaction). Residual TPH contents correlated significantly positively with the archaeal abundance and negatively with the abundance of *gtlA* gene; no significant correlations with the other microbial abundance measurements were noted (Table 1).

**Table 1:** Bacterial, archaeal and fungal abundances and potential aerobic bacterial respiration determined through qPCR in the contaminated soil at initial conditions (t0) and after 15 weeks without fertilization at 10 (UNF10) and 20 °C (UNF20) and with NPK fertilization at 10 (NPK10) and 20 °C (NPK20)

	Experimental treatments^a^					One-way ANOVA^a^	MANOVA^b^	Correlations with TPH contents^c^
	t0	UNF10	UNF20	NPK10	NPK20	*F* (*p* value)		*R* (*p* value)
*Bacteria* (B)^d^	6.06 a	6.00 a	6.03 a	6.17 b	6.17 b	*6.90* (0.0007)	F***	− 0.2568 (0.3554)
*Archaea* (A)^d^	4.40 b	3.99 b	4.02 b	3.04 a	2.98 a	*34.67* (< 0.0001)	F***	*0.6531* (0.0083)
*Fungi* (F)^e^	2.76 b	2.98 b	3.56 c	3.08 bc	2.02 a	*17.10* (< 0.0001)	F***, *F* × T***	0.1686 (0.5481)
A/B	0.73 b	0.66 b	0.67 b	0.49 a	0.48 a	*40.67* (< 0.0001)	F***	*0.6432* (0.0097)
F/B	0.45 b	0.50 b	0.59 c	0.50 bc	0.33 a	*17.77* (< 0.0001)	F***, *F* × T***	0.1779 (0.5259)
*gtlA* ^f^	2.85 a	2.74 a	3.06 b	3.06 b	3.34 c	*46.79* (< 0.0001)	F***, T***	**−** *0.6201* (0.0137)

^a^For one-way ANOVA of each variable, *F* values in italics denote statistical significance (*p* ≤ 0.05); for Tukey’s HSD tests, mean values followed by different letters are significantly different (*p* ≤ 0.05)

^b^For MANOVA, significance levels of the factors fertilization (F) and temperature (T) as well as their interaction for each variable are shown at *p ≤ 0.05, **p ≤ 0.01, and ***p ≤ 0.001

^c^Results of correlation analyses (Pearson method) between contents of total petroleum hydrocarbons (TPH) and the different abundance measurements considering the five treatments; *R* values in italics denote statistical significance (*p* ≤ 0.05)

^d^Log 16S rRNA gene copy number (μg extracted soil DNA)^−1^

^e^Log 18S rRNA gene copy number (μg extracted soil DNA)^−1^

^f^Log *gtlA* gene copy number (μg extracted soil DNA)^−1^

### Diversity and composition of prokaryotic community

**Fig. 1:**
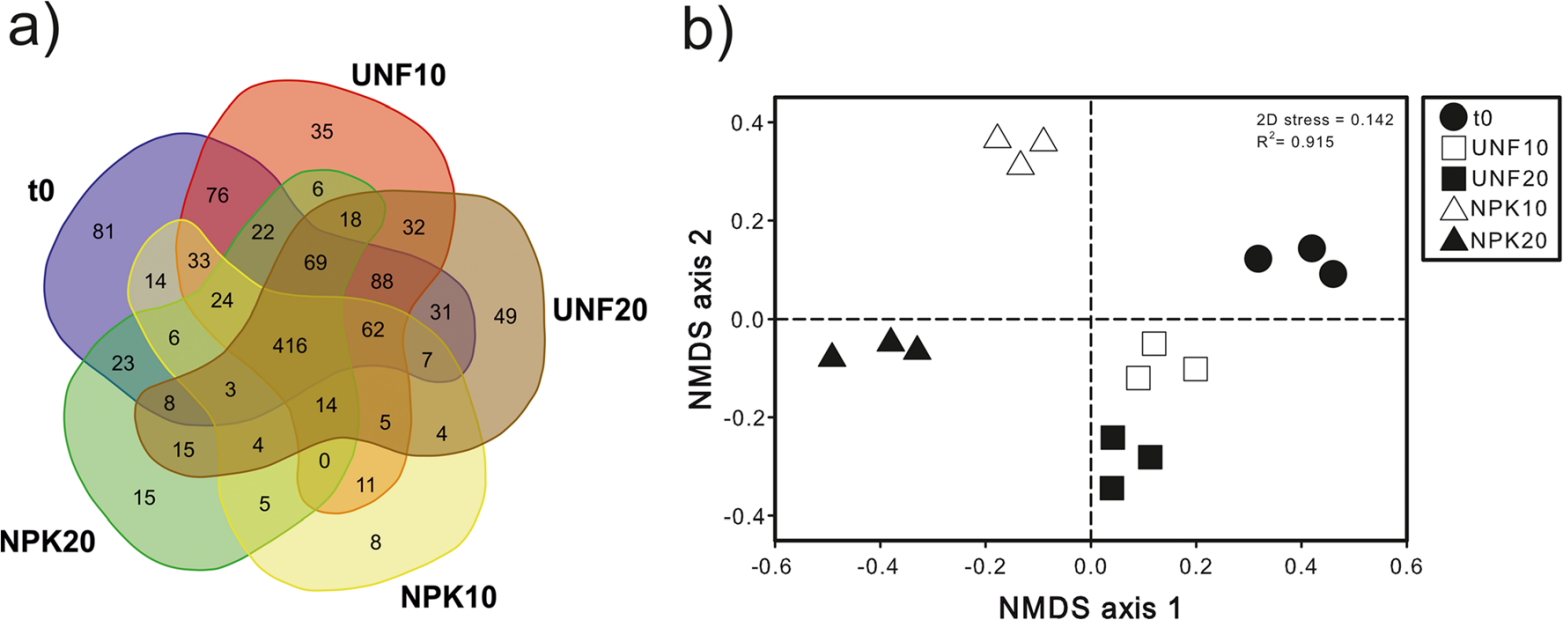
Venn diagram showing the number of unique and shared bacterial OTUs between the contaminated soil at initial conditions (t0) and after 15 weeks without and with fertilization at 10 and 20 °C (UNF10, UNF20, NPK10, NPK20) (**a**). Non-metric multidimensional scaling (NMDS) ordination based on Bray-Curtis similarities of OTU-based bacterial community structures found in the different soil treatments (**b**)

Illumina analysis of prokaryotic communities yielded a total of 1,632,143 paired-end high-quality 16S rRNA sequences across the 15 libraries analyzed. 95.5% of those reads were successfully mapped back and distributed among the 1258 OTUs generated by UPARSE-OTU algorithm. Of these OTUs, 1184 were classified as *Bacteria* (using a 50% confidence threshold). Regardless of their relative abundances, 35.1% (416) of total OTUs were found in all investigated soil samples (t0, UNF10, UNF20, NPK10, NPK20) (Fig. 1a). The control soil (t0) had the highest number of unique OTUs (6.8% of total OTUs), while the lowest one (0.7% of total OTUs) was found after 15 weeks in the NPK treatment at 10 °C. Richness as well as ACE and Shannon indices was significantly higher in the unfertilized soil with respect to the fertilized soil at both temperatures (Table 2). Increased temperature determined a decrease in richness in the unfertilized soil and an increase for the Shannon index in the fertilized soil. In the case of evenness, increased values were found both in the absence/presence of nutrients at both temperatures with respect to the soil at initial conditions. The interaction fertilization and temperature was significant for all the diversity measurements except evenness. Richness and ACE always correlated significantly positively with the residual TPH content, while evenness did so negatively (Table 2).

**Table 2:** Richness and diversity characteristics of bacterial and fungal communities in the contaminated soil at initial conditions (t0) and after 15 weeks without fertilization at 10 °C (UNF10) and 20 °C (UNF20) and with NPK fertilization at 10 °C (NPK10) and 20 °C (NPK20)

	Experimental treatments^a^					One-way ANOVA^a^	MANOVA^b^	Correlations with TPH contents^c^
	t0	UNF10	UNF20	NPK10	NPK20	*F* (*p* value)		*R* (*p* value)
Bacterial communities								
Richness	717.0 c	687.7 c	642.7 b	439.3 a	483.3 a	*172.00* (< 0.0001)	F***, *F* × T***	*0.5994* (0.0182)
Shannon index	3.62 b	3.97 c	4.04 c	2.89 a	3.63 b	*252.63* (< 0.0001)	F***, T***, *F* × T***	− 0.0883 (0.7545)
ACE	868.6 b	842.5 b	769.4 b	575.4 a	616.3 a	*37.94* (< 0.0001)	F***, *F* × T***	*0.6166* (0.0144)
Evenness	0.501 a	0.513 b	0.518 b	0.517 b	0.525 c	*88.15* (< 0.0001)	F***, T***	**−** *0.9663* (< 0.0001)
Fungal communities								
Richness	51.3 bc	55.0 c	38.0 a	44.3 ab	47.7 bc	*11.45* (0.0009)	F**, *F* × T**	0.3113 (0.2587)
Shannon index	1.69 bc	2.26 c	1.41 b	1.35 b	0.63 a	*19.47* (0.0001)	F***, T***	0.4906 (0.0634)
ACE	63.5 a	74.2 a	66.1 a	55.6 a	60.6 a	2.72 (0.091)		0.0353 (0.9005)
Evenness	0.552 ab	0.559 b	0.559 b	0.550 ab	0.531 a	*5.43* (0.0138)	F**	0.3598 (0.1878)

^a^For one-way ANOVA of each variable, *F* values in italics denote statistical significance (*p* ≤ 0.05); for Tukey’s HSD tests, mean values followed by different letters are significantly different (*p* ≤ 0.05)

^b^For MANOVA, significance levels of the factors fertilization (F) and temperature (T) as well as their interaction for each variable are shown at *p ≤ 0.05, **p ≤ 0.01, and ***p ≤ 0.001

^c^Results of correlation analysis (Pearson method) between contents of total petroleum hydrocarbons (TPH) and the different richness and diversity measurements considering the five treatments; *R* values in italics denote statistical significance (*p* ≤ 0.05)

NMDS ordination of OTU-based structure of bacterial communities showed that the factor determining sample ordination over the axis 1 was the presence/absence of NPK fertilization; over the axis 2, fertilized samples were clustered according to temperature, while the unfertilized soil samples at 10 and at 20 °C were grouped together and separated from the soil at initial conditions (Fig 1b). PERMANOVA (*F* = 244.7; *p* = 0.0001), ANOSIM (*r* = 1; *p* = 0.0001), and MRPP (*δ* = 0.058; *p* = 0.0001) confirmed the significance of this NMDS ordination. The factors fertilization (PERMANOVA, *F* = 355.6, *p* = 0.0002; ANOSIM, *r* = 1, *p* = 0.0099; MRPP, *δ* = 0.309, *p* = 0.0022) and temperature (PERMANOVA, *F* = 184.4, *p* = 0.0002; ANOSIM, *r* = 1, *p* = 0.012; MRPP, *δ* = 0.38, *p* = 0.0052) as well as their interaction (PERMANOVA, *F* = 165.6, *p* = 0.0003) had a significant effect on the structure of bacterial community. In concordance with NMDS, pairwise SIMPER analysis demonstrated that the highest average dissimilarity in the structure of bacterial community was found between the soil at initial conditions and the fertilized soil at 20 °C (Supplemental Table S1). According to the Mantel test, shifts in the OTU-based structure of bacterial community were significantly explained by variations in TPH contents in all studied soil samples (*r* = 0.5105; *p =* 0.0001).

**Fig. 2:**
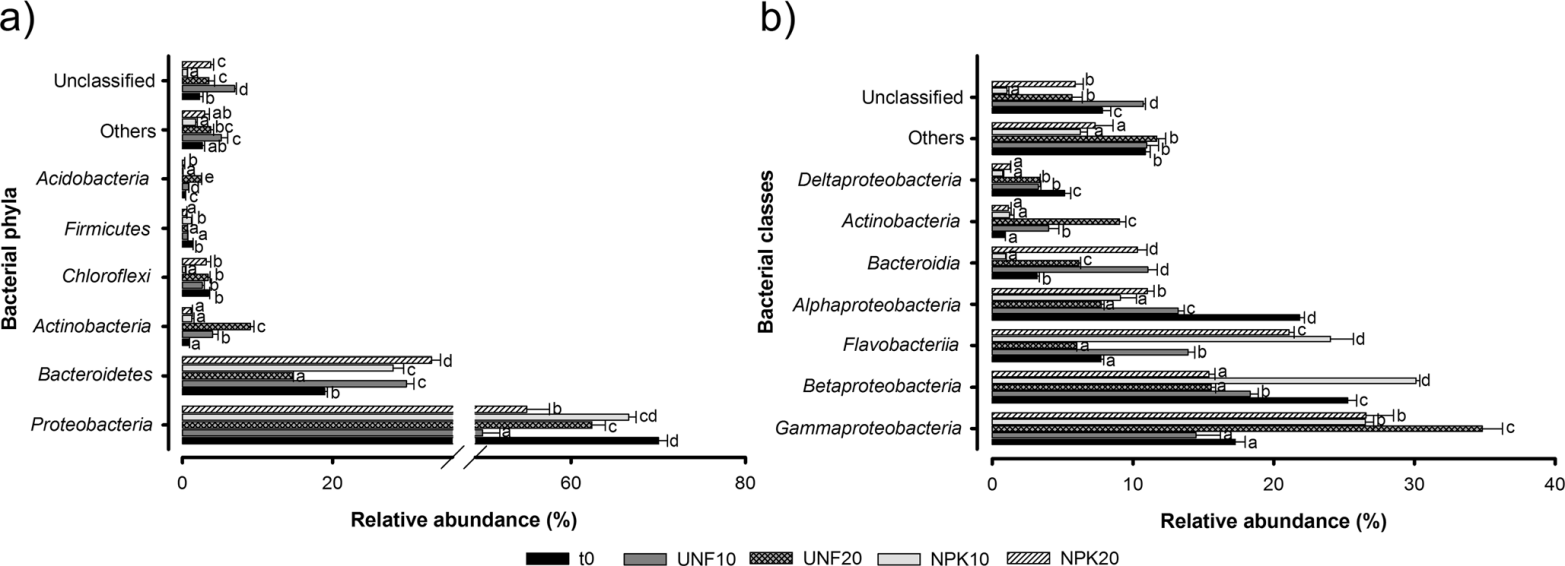
Relative abundance of the main bacterial phyla (**a**) and classes (**b**) found in the contaminated soil at initial conditions (t0) and after 15 weeks without and with fertilization at 10 and 20 °C (UNF10, UNF20, NPK10, NPK20). The sum for each treatment of the relative abundances obtained for the different taxonomic groups and unclassified sequences is equal to 100. For each bacterial taxonomic group, mean values followed by different letters are significantly different (*p* ≤ 0.05) according to Tukey’s HSD test. Bars represent standard deviation

At taxonomic level, total bacterial diversity was distributed among 27 different phyla. *Proteobacteria* and *Bacteroidetes* were clearly the predominant ones since they accounted for more than 75.1% of the total sequences (Fig. 2a, Supplemental Table S2). The shifts in the relative abundance of these phyla responded to a significant interaction of the factors fertilization and temperature (as shown by statistical multivariate analyses of OTU-based structure of bacterial communities). A more detailed analysis of *Proteobacteria* phylum at class level evidenced that *Gammaproteobacteria* were significantly more abundant in the UNF20 treatment and with NPK fertilization at both temperatures and always significantly negatively correlated with TPH contents (Fig. 2b, Supplemental Table S2). Instead, *Betaproteobacteria*, *Alphaproteobacteria*, and *Deltaproteobacteria* were detected to a significantly lower extent after 15 weeks, regardless of the absence/presence of nutrients and temperature, with respect to t0 (with the exception of *Betaproteobacteria* in the NPK10 treatment), and their relative abundances correlated significantly positively with TPH contents. Within *Bacteroidetes* phylum, both *Flavobacteriia* and *Bacteroidia* classes were increased after 15 weeks with and without fertilization with respect to t0 (with the exception of UNF20 for *Flavobacteriia* and NPK10 for *Bacteroidia*), but only changing relative abundances of *Bacteroidia* were significantly negatively explained by variations in TPH contents (Fig. 2b, Supplemental Table S2). Also within *Bacteroidetes* phylum, a significant decrease in the relative abundance of *Sphingobacteriia* had generally occurred after 15 weeks and was significantly positively correlated with TPH contents. Among the other phyla detected, relative abundances of *Actinobacteria* were significantly increased in the unfertilized soil at both temperatures (Supplemental Table S2). Top 30 OTUs (from a total of 1258 OTUs that comprised bacterial communities) reported from SIMPER analysis contributed to 69.9% of the total dissimilarity in bacterial community structures (Supplemental Table S3). Considering the two classes negatively correlating with TPH contents, gammaproteobacterial OTUs belonging to genera such as *Pseudomonas*, *Lysobacter*, *Povalibacter*, *Solimonas*, and *Pseudoxanthomonas* as well as bacteroidial OTUs classified at genus level as *Mangrovibacterium*, *Paludibacter*, *Petrimonas*, and *Mariniphaga* greatly contributed to dissimilarity (Supplemental Table S3).

**Fig. 3:**
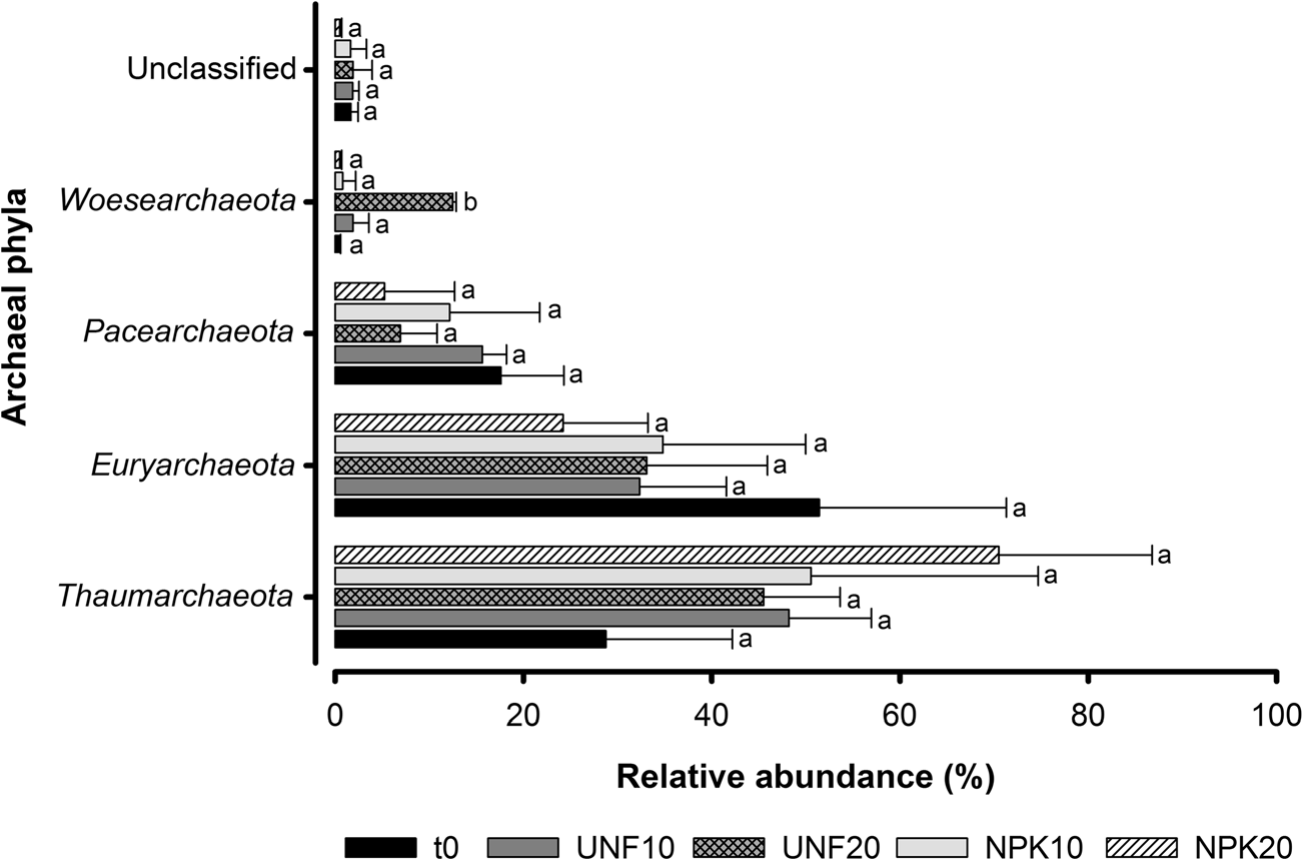
Relative abundance of the archaeal phyla found in the contaminated soil at initial conditions (t0) and after 15 weeks without and with fertilization at 10 and 20 °C (UNF10, UNF20, NPK10, NPK20). The sum for each treatment of the relative abundances obtained for the different phyla and unclassified sequences is equal to 100. For each archaeal phylum, values followed by different letters are significantly different (*p* ≤ 0.05) according to Tukey’s HSD test. Bars represent standard deviation

In the case of archaeal community, 65 of the initial 1258 prokaryotic OTUs were classified as *Archaea*, which grouped a total of 7440 sequences. *Thaumarchaeota*, *Euryarchaeota*, and *Pacearchaeota* phyla clearly dominated archaeal community and significant differences in their relative abundances were not found after 15 weeks (Fig. 3). Only the minor phylum *Woesearchaeota* had increased in the treatment UNF20 with respect to all the other treatments. *Nitrosopumilales* and *Nitrososphaerales* classes dominated among *Thaumarchaeota*, while *Methanomicrobia* and *Thermoplasmata* dominated among *Euryarchaeaota*. Significant differences in the relative abundance of the major archaeal classes were not detected in any of the treatments (data not shown).

### Diversity and composition of fungal community

**Fig. 4:**
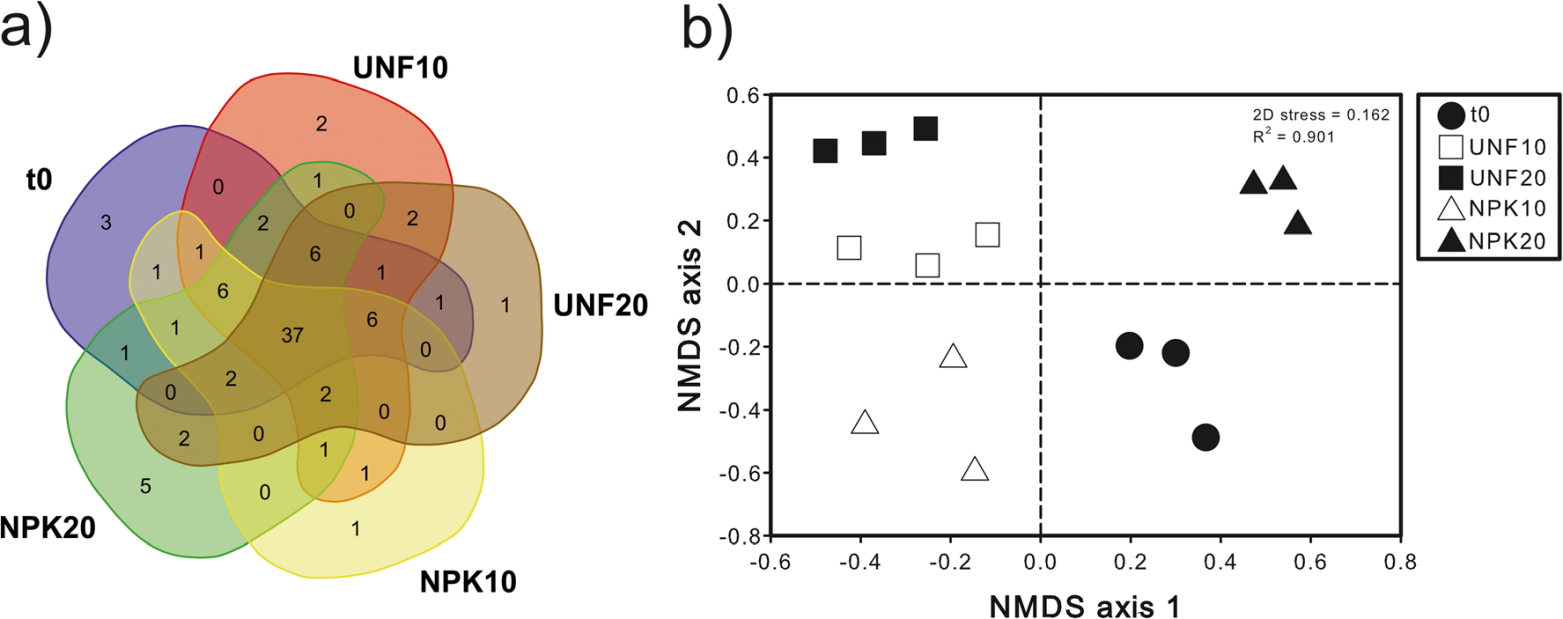
Venn diagram showing the number of unique and shared fungal OTUs between the contaminated soil at initial conditions (t0) and after 15 weeks without and with fertilization at 10 and 20 °C (UNF10, UNF20, NPK10, NPK20) (**a**). Non-metric multidimensional scaling (NMDS) ordination based on Bray-Curtis similarities of OTU-based fungal community structures found in the different soil treatments (**b**)

A total of 1,312,768 paired-end high-quality ITS sequences were obtained after fungal Illumina-based analysis. Most of these reads (99.9%) were mapped back and distributed among the 87 OTUs that comprised fungal community, all of which could be successfully classified as *Fungi*. Venn diagram showed that, irrespectively of their relative abundances, 37 of those 87 OTUs were shared among all the samples, being the number of unique OTUs in each treatment very low (Fig. 4a). Regarding the different diversity measurements, fungal richness was significantly influenced by the interaction fertilization and temperature (*F* = 24.97; *p* = 0.0011); a higher temperature determined a decrease in fungal richness in unfertilized soil, but an increase in fungal richness in NPK-fertilized soil. The Shannon index was lower with fertilization, regardless of temperature, with respect to t0; significantly lower Shannon index values were observed in unfertilized and NPK-fertilized soil at 20 °C than at 10 °C (Table 2). No significant correlations (*p* > 0.05) were found between TPH contents and the different fungal diversity and richness measurements (Table 2).

PERMANOVA (*F* = 17.27; *p* = 0.0001), ANOSIM (*r* = 0.9941; *p* = 0.0001), and MRPP (*δ* = 0.30; *p* = 0.0001) demonstrated that OTU-based structure of fungal communities varied among the studied soil samples, which was graphically evidenced through the NMDS ordination of soil samples (Fig. 4b). Although NMDS clustered unfertilized soil at 10 and 20 °C, both fertilization (PERMANOVA, *F* = 29.17, *p* = 0.0002; ANOSIM, *r* = 0.6871, *p* = 0.0034; MRPP, *δ* = 0.54, *p* = 0.0022) and temperature (PERMANOVA, *F* = 16.90, *p* = 0.0001; ANOSIM, *r* = 0.3574, *p* = 0.0211; MRPP, *δ* = 0.63, *p* = 0.0159), as well as their interaction (PERMANOVA, *F* = 11.96, *p* = 0.0004), influenced significantly fungal community structure. Pairwise SIMPER analysis highlighted that the structure of fungal community differed the most between unfertilized soil at 20 °C and fertilized soil at 10 °C (Supplemental Table S4). The treatment showing the highest average dissimilarity in the fungal community structure compared to t0 was unfertilized soil at 20 °C. According to the Mantel test, shifts in the structure of OTU-based structure of fungal community were significantly explained by the TPH contents (*r* = 0.4453; *p =* 0.0006).

**Fig. 5:**
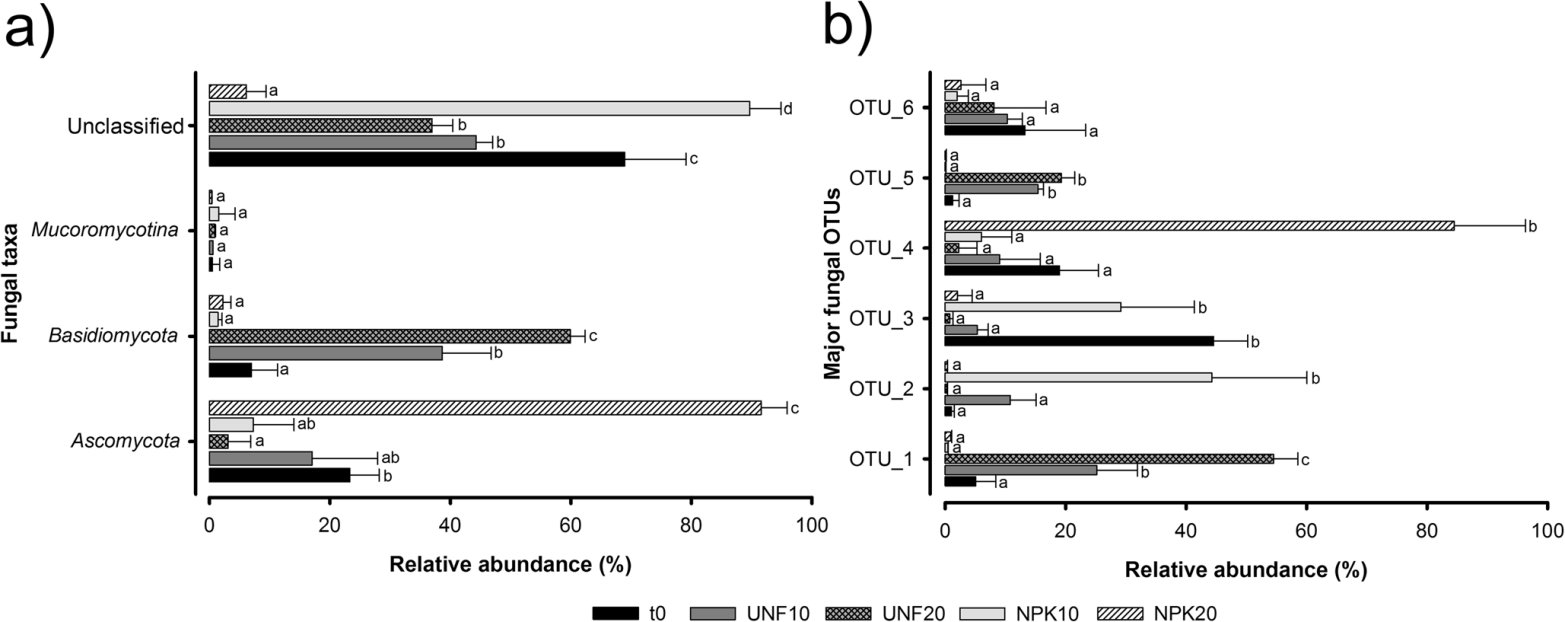
Relative abundance of the fungal phyla (*Ascomycota* and *Basidiomycota*) and subphylum (*Mucoromycotina*) (**a**) and major OTUs (**b**) found in the contaminated soil at initial conditions (t0) and after 15 weeks without and with fertilization at 10 and 20 °C (UNF10, UNF20, NPK10, NPK20). The sum for each treatment of the relative abundances obtained for the different taxonomic groups and unclassified sequences as well as OTUs is equal to 100. For each taxonomic group or OTU, values followed by different letters are significantly different (*p* ≤ 0.05) according to Tukey’s HSD test. Bars represent standard deviation

Overall composition of fungal communities across the 15 libraries analyzed was distributed among two phyla and one subphylum (Fig. 5a). *Ascomycota* (the proportion of classified sequences in this phylum ranged from 0.6 to 95.3%) and *Basidiomycota* (0.7–62.7%) were the predominant ones, although their relative abundances greatly differed across the libraries (Fig. 5a, Supplemental Table S5). A very large proportion of sequences were unclassified (using a 50% confidence threshold on RDP classifier) at phylum (ranging from 2.9 to 94.0%), class (70.5–98.8%), and genus levels (77.1–98.8%). To decipher the reason behind these huge proportions of unclassified sequences, the distribution of the total number of sequences among the 84 OTUs comprising fungal community in the 15 libraries was analyzed, discovering that the top six (OTU1-6) most abundant OTUs concentrated more than 76.1% of the total number of sequences (Fig. 5b) and these OTUs were not successfully classified at phylum, class, or genus levels (with the exception of OTU1 and 4 at phylum level) (Supplemental Table S6). Based on the taxonomic information obtained from the successfully classified OTUs, we observed an increase of the relative abundance of *Ascomycota* in the fertilized soil at 20 °C. Instead, *Basidiomycota* were found to a significantly higher extent in the unfertilized soil at 20 °C (Fig. 5a, Supplemental Table S5). Among the different classes identified, significant changes in their relative abundances were not noticed (Supplemental Table S5). None of the taxa, at phylum or class levels, significantly correlated with TPH contents (Supplemental Table S5). SIMPER analysis showed that top 20 OTUs contributed to 91.5% of the dissimilarity in fungal community structures (Supplemental Table S6). Some of these 20 OTUs were successfully classified at genus level as *Cylindrobasidium* (OTU14), *Fomitopsis* (OTU15), *Candida* (OTU22), and *Penicillium* (OTU25), but none of them were significantly negatively correlated with TPH contents. However, according to the Mantel test, shifts in TPH contents could be significantly explained by the variations in the relative abundance of these 20 OTUs (*r* = 0.4528; *p =* 0.0011).

### Summarizing the effects of fertilization and temperature on characteristics of soil microbial communities during bioremediation

The factor fertilization (absence/presence of nutrients) during soil bioremediation was significant for the abundance of bacterial, archaeal, and fungal communities and copy number of *gtlA* gene, diversity characteristics of bacterial and fungal communities as well as for the OTU-based structure of bacterial and fungal abundances (Tables 1 and 2). In comparison, the significant effect of the factor temperature was more limited, only determining significant shifts in the abundance of *gtlA* gene, fungal Shannon index, and structure of bacterial and fungal communities. However, the interaction of factors fertilization and temperature significantly influenced fungal abundances, bacterial richness and diversity, fungal richness, and structures of bacterial and fungal communities.

## Discussion

The integrative study of microbial communities involved in soil bioremediation is important to identify the specific microbial characteristics that determine improved decontamination rates, which can be further used to optimize the existing bioremediation strategies and to develop new ones (Stenuit et al. 2008). To the best our knowledge, our study is one of the first reports describing the effect of temperature and fertilization on abundance, diversity, and composition of bacterial, archaeal, and fungal communities in a long-term hydrocarbon-contaminated soil.

Regardless of temperature, fertilization resulted in an increase in bacterial abundance compared to natural attenuation after 15 weeks. These findings, as well as those obtained for fungal community (significant increase in fungal abundance at 10 °C), concur with the data obtained through the PLFA-based analysis (Mair et al. 2013), thus indicating that most of the microorganisms targeted through qPCR are living microorganisms (Blagodatskaya and Kuzyakov 2013). In general, higher microbial abundances are expected after fertilizer addition since increased levels of nutrients stimulate bacterial growth, especially that of r-strategists (Girvan et al. 2004; Margesin et al. 2003; Wu et al. 2016). Increased bacterial abundances were not significantly correlated with TPH removal rates. However, abundances of *gtlA* gene, as functional marker of aerobic bacterial activity (respiration), were significantly negatively correlated with residual TPH contents, especially in the case of fertilization; the same was observed when soil respiration was determined (Mair et al. 2013). These data suggest that improved decontamination rates could be explained to a higher extent by the stimulation of the activities of indigenous soil microorganisms than by the enhancement of microbial abundances (Bastida et al. 2016).

In the present study, bacterial richness (richness and ACE index) and diversity (Shannon index) values had not significantly decreased after 15 weeks of bioremediation, despite significant TPH removal rates, which is in contrast to our initial hypothesis. Previous studies reported case-sensitive responses of bacterial communities, in terms of diversity, to changing concentrations of hydrocarbons (Sutton et al. 2013). Our data also show that the homogeneity of soil bacterial community increased (higher evenness values) in parallel with TPH removal. This may be the result of the existence of a well-established bacterial community in the soil under study, adapted to use the existing contaminants as source of carbon and energy. On the other hand, TPH contents significantly explained the shifts in bacterial community composition (supporting our initial hypothesis). The OTU-based structure of bacterial communities varied as a consequence of temperature and of the absence/presence of fertilization. *Proteobacteria* and *Bacteroidetes* were the predominant phyla, which have commonly been found in hydrocarbon-contaminated soils (Shahi et al. 2016a, b; Sutton et al. 2013). Although bacterial taxonomic composition varied among treatments, we found statistical evidences that the same bacterial groups, namely *Gammaproteobacteria* and *Bacteroidia* classes, were involved in the TPH removal in both natural attenuation and biostimulation. The PLFA-based analysis also evidenced the key role of Gram-negative bacteria (such as *Gammaproteobacteria* and *Bacteroidia*) on the bioremediation of the studied soil (Mair et al. 2013). A number of studies reported the active involvement of *Gammaproteobacteria* in the degradation of petroleum hydrocarbons after biostimulation approaches; the “gamma-shift” is a well-known phenomenon characterizing bacterial communities of soils with changing hydrocarbon concentrations and is a result of the their enrichment following hydrocarbon contamination (Dong et al. 2015; Labbé et al. 2007; Militon et al. 2010). Members of this taxonomic group, such as *Pseudomonas* spp. (in our study, OTUs belonging to this genus contributed the most to bacterial community composition dissimilarity), are effective alkane and aromatic hydrocarbon degraders in petroleum-contaminated soils (Qin et al. 2013).

Archaeal community across the 15 libraries analyzed in this study only represented between 0.05 and 3.2% of the total prokaryotic community, a proportion that is much lower than that described for other soils (Wang et al. 2015). We see this finding in connection with the very low amount of archaeal sequences that were found in most of the samples of our Illumina-based analysis of soil prokaryotic communities. Kim et al. (2018) also found a very low proportion of archaeal sequences when analyzing the prokaryotic community (at a comparable sequencing depth) during bioremediation of hydrocarbon-contaminated soils. In our study, archaeal abundances significantly positively correlated with TPH contents, which evidenced that archaeal community was not involved in bioremediation at the time point considered (after 15 weeks). We also noticed a decrease of archaeal abundances in the presence of fertilization (regardless of temperature) after 15 weeks, which could be related to the adaptation of the archaeal community to oligotrophic nutrient conditions prevailing in the studied soil and its poor competitive ability in respect to groups of r-strategist bacteria that were favored, in terms of abundance, by fertilization (Karlsson et al. 2012). In taxonomic terms, we did not find statistical evidences of the involvement of the different archaeal phyla or classes identified in the bioremediation process. In concordance with our data, members of the archaeal phyla *Thaumarchaeota* and *Euryarchaeota* have shown to commonly inhabit hydrocarbon-rich environments (Jeanbille et al. 2016; Joshi et al. 2014; Sarkar et al. 2016); however, archaeal communities play a noteworthy role on hydrocarbon degradation only under anaerobic conditions (Das and Kazy 2014; Fowler et al. 2016). In our study, therefore, the lack of a remarkable role of archaeal communities in bioremediation can be a consequence of the aerobic conditions that characterized the feasibility study.

Contrary to our initial hypothesis, increased TPH removal rates were not concomitant with higher fungal abundances and lower fungal richness and diversities in our study. Our preliminary expectations were based on studies such as that of Covino et al. (2016), which showed that increased fungal abundances (as determined by ergosterol measurement) and lower fungal richness and the Shannon index (as determined by Illumina amplicon sequencing) values went in parallel with decreased concentrations of hydrocarbons during the bioremediation of a long-term oil-contaminated soil through biostimulation for 30 and 60 days using a lignocellulose mixture. In our case, the dynamics of fungal abundance, richness, and the Shannon index among the different treatments seemed to respond to a significant interaction of the factors fertilization and temperature, probably because these dynamics were driven by variations in other soil physicochemical properties, in place of TPH contents. Instead, shifts in the structure of fungal communities among the investigated treatments were significantly explained by changing TPH contents. Interestingly, Zhou et al. (2017), investigating the fungal communities inhabiting a set of soils contaminated with different contents of PAHs, also found a significant correlation of PAH contents with fungal community structure, but not with fungal diversity (Shannon index), and explained this as a consequence of the lower sensitivity of fungal communities to the prevailing contaminants in comparison with bacteria.

The most remarkable finding obtained from taxonomic compositional analysis of fungal communities was the high proportion of unclassified sequences at the different taxonomic levels in each of the treatments. We assigned OTUs to taxa by using the RDP classifier (Wang et al. 2007) against the UNITE database with a 50% confidence threshold, as previously reported (Barberan et al. 2015; Siles and Margesin 2017); and although other tools (sintax command of USEARCH pipeline) and training sets (WARCUP (Deshpande et al. 2016)) were also applied to try to improve the proportion of classified reads, similar rates of classified sequences were obtained. These results can have two possible explanations: (i) the sequence length may have been too short for accurate classification, or (ii) the reference database may not be complete enough to include all the taxonomic information at the different taxonomic levels and some comparative elements were missing for the classification of the complete fungal diversity (Rachid et al. 2013). In our case, since we obtained in previous works (Siles and Margesin 2016, 2017) much lower proportion of unclassified sequences at the different taxonomic levels using the same approaches as those utilized here for taxonomic affiliation of reads with a similar length, and since all the OTUs integrating fungal community were successfully classified at kingdom level (*Fungi*), we explain the high proportion of sequences without an effective taxonomic classification as a consequence of the presence of a high proportion of undescribed fungal diversity in the studied soil. Former studies have also interpreted high proportions of unclassified sequences as the result of the affiliation of those sequences to as-yet-uncultured, unrecognized, or novel microbial groups (Sun et al. 2014; Sutton et al. 2013). Since we found significant correlations between the top 20 OTUs contributing to dissimilarity in fungal community structures among the studied treatments and TPH contents, the potentially new fungal species inhabiting this soil may have contributed to some extent to bioremediation.

In conclusion, we here demonstrated that the bioremediation of an Alpine long-term hydrocarbon-contaminated soil after 15 weeks of natural attenuation or NPK fertilization at 10 and 20 °C was mainly driven by changes in bacterial and fungal community structures. Changing features of archaeal communities, in general, and shifts in the abundances and diversities of bacterial and fungal communities were not the decisive factor in soil bioremediation. Bacterial *Gammaproteobacteria* and *Bacteroidia* classes were related to TPH removal rates in both unfertilized and fertilized treatments. Fungal OTUs related to the bioremediation process were taxonomically affiliated to undescribed/novel groups. Fungal abundance and richness, bacterial richness, and diversity as well as OTU-based structures of bacterial and fungal communities were significantly influenced by the interaction of temperature and fertilization. Further functional studies (e.g., Geochip- or transcriptomic-based) are needed to deeply decipher the response of indigenous microbial communities mediating the bioremediation of hydrocarbon-contaminated soils in terms of activity.

## Electronic supplementary material

ESM 1 (PDF 123 kb)
