# Supplementary material for: Insights into microbial communities mediating the bioremediation of hydrocarbon-contaminated soil from an Alpine former military site
Source: Appl Microbiol Biotechnol. 2018 Mar 29;102(10):4409–21. doi: 10.1007/s00253-018-8932-6 (PMC5932094; doi:10.1007/s00253-018-8932-6)
Supplement: Supplementary file 1 — (PDF 123 kb) [file 253_2018_8932_MOESM1_ESM.pdf]

# SUPPLEMENTARY MATERIAL

## APPLIED MICROBIOLOGY AND BIOTECHNOLOGY

# Insights into microbial communities mediating the bioremediation of hydrocarbon-contaminated soil from an Alpine former military site

José A. Siles[1] ● Rosa Margesin[1,*]

[1]Institute of Microbiology, University of Innsbruck, Technikerstrasse 25, A-6020 Innsbruck, Austria

[*]Corresponding author. *E-mail address*: rosa.margesin@uibk.ac.at;*Tel.*:+43 512 507-51230; *Fax*: +43 512 507-51240.

**The supplementary material includes Supplemental Tables S1-S6**

**Supplemental Table S1**. Average dissimilarity values obtained after pairwise SIMPER (similarity percentage) analysis of OTU-based bacterial community structures in the contaminated soil at initial conditions (t0) and after 15 weeks without fertilization at 10 (UNF10) and 20 °C (UNF20) and with NPK fertilization at 10 (NPK10) and 20°C (NPK20).

| | t0 | UNF10 | UNF20 | NPK10 | NPK20 |
|---|---|---|---|---|---|
| t0 | | | | | |
| UNF10 | 36.78 | | | | |
| UNF20 | 48.60 | 39.84 | | | |
| NPK10 | 56.23 | 57.81 | 63.15 | | |
| NPK20 | 65.11 | 55.75 | 59.22 | 55.03 | |

**Supplemental Table S2**. Relative abundances (%) of the most abundant bacterial phyla and classes found in the contaminated soil at initial conditions (t0) and after 15 weeks without fertilization at 10 (UNF10) and 20 °C (UNF20) and with NPK fertilization at 10 (NPK10) and 20°C (NPK20). For each taxonomic group, mean values followed by different letters are significantly different ($p \leq 0.05$) according to Tukey's HSD test. Results of correlation analysis (Pearson method) between contents of total petroleum hydrocarbons (TPH) and relative abundances of the different taxonomic groups considering the five treatments are also given; R values in bold denote statistical significance ($p \leq 0.05$).

| **Taxa** | | **Experimental treatments** | | | | | **Correlations with TPH contents** |
|---|---|---|---|---|---|---|---|
| | | **t0** | **UNF10** | **UNF20** | **NPK10** | **NPK20** | **R (*p-value*)** |
| *Proteobacteria* | | 70.04 **d** | 49.84 **a** | 62.40 **c** | 66.60 **cd** | 54.91 **b** | **0.6366** (0.0107) |
| *Proteobacteria* classes | *Gammaproteobacteria* | 17.27 **a** | 14.48 **a** | 34.82 **c** | 26.52 **b** | 26.55 **b** | -**0.5100** (0.0491) |
| | *Betaproteobacteria* | 25.26 **c** | 18.34 **b** | 15.56 **a** | 30.12 **d** | 15.40 **a** | **0.5614** (0.0294) |
| | *Alphaproteobacteria* | 21.84 **d** | 13.21 **c** | 7.74 **a** | 9.10 **a** | 11.02 **b** | **0.8329** (0.0001) |
| | *Deltaproteobacteria* | 5.14 **c** | 3.27 **b** | 3.34 **b** | 0.75 **a** | 1.28 **a** | **0.7531** (0.0012) |
| *Bacteroidetes* | | 18.97 **b** | 29.84 **c** | 14.65 **a** | 28.08 **c** | 33.19 **d** | -0.4875 (0.0653) |
| *Bacteroidetes* classes | *Flavobacteriia* | 7.73 **a** | 13.92 **b** | 5.98 **a** | 24.03 **d** | 21.08 **c** | -0.4801 (0.0701) |
| | *Bacteroidia* | 3.19 **b** | 11.06 **d** | 6.14 **c** | 0.95 **a** | 10.31 **d** | **-0.5365** (0.0392) |
| | *Sphingobacteriia* | 3.36 **c** | 1.62 **b** | 1.45 **b** | 0.22 **a** | 0.20 **a** | **0.8703** (0.0000) |
| | *Cytophagia* | 0.15 **a** | 0.07 **a** | 0.03 **a** | 2.59 **b** | 0.10 **a** | -0.0119 (0.9663) |
| *Actinobacteria* | | 0.87 **a** | 4.01 **b** | 9.05 **c** | 1.24 **a** | 1.15 **a** | -0.2658 (0.3383) |
| *Class* level | *Actinobacteria* | 0.87 **a** | 4.01 **b** | 9.05 **c** | 1.24 **a** | 1.15 **a** | -0.2660 (0.3379) |
| *Chloroflexi* | | 3.47 **b** | 2.65 **b** | 3.42 **b** | 0.41 **a** | 3.16 **b** | 0.1120 (0.6911) |
| *Chloroflexi* classes | *Anaerolineae* | 3.41 **b** | 2.59 **b** | 3.32 **b** | 0.40 **a** | 3.14 **b** | 0.1075 (0.7029) |
| *Firmicutes* | | 1.29 **b** | 0.70 **a** | 0.67 **a** | 1.21 **b** | 0.55 **a** | **0.7872** (0.0005) |
| *Firmicutes* classes | *Clostridia* | 0.74 **b** | 0.58 **ab** | 0.48 **a** | 1.19 **c** | 0.52 **a** | 0.2622 (0.3452) |
| *Acidobacteria* | | 0.41 **c** | 0.82 **d** | 2.52 **e** | 0.07 **a** | 0.30 **b** | -0.1762 (0.5298) |
| Other phyla | | 2.64 **ab** | 5.18 **c** | 3.77 **bc** | 1.76 **a** | 2.98 **ab** | -0.1977 (0.4800) |
| | Other classes | 3.22 **b** | 6.11 **c** | 6.40 **c** | 1.85 **a** | 3.35 **b** | -0.2030 (0.4680) |
| Unclassified (phylum level) | | 2.29 **b** | 6.95 **d** | 3.52 **c** | 0.63 **a** | 3.75 **c** | -0.2567 (0.3557) |
| | Unclassified (class level) | 7.82 **c** | 10.73 **d** | 5.67 **b** | 1.04 **a** | 5.89 **b** | 0.2100 (0.4525) |

**Supplemental Table S3**. Taxonomic classification of the top 30 OTUs contributing to dissimilarities (similarity percentage analysis) in bacterial community structures among the five treatments (t0, UNF10, UNF20, NPK10, NPK20) using a 50% confidence threshold (ribosomal database project taxonomic classifier). OTUs were ordered in decreasing order of dissimilarity contribution.

| OTU number | Average dissimilarity | Dissimilarity contribution (%) | Cumulative diss. contribution (%) | Phylum | Class | Genus |
|---|---|---|---|---|---|---|
| OTU_3 | 4.73 | 8.79 | 8.79 | *Bacteroidetes* | *Flavobacteriia* | *Lutibacter* |
| OTU_1 | 3.91 | 7.27 | 16.06 | *Proteobacteria* | *Betaproteobacteria* | Unclassified |
| OTU_5 | 2.76 | 5.13 | 21.19 | *Proteobacteria* | *Alphaproteobacteria* | *Caulobacter* |
| OTU_7 | 2.53 | 4.70 | 25.89 | *Proteobacteria* | *Gammaproteobacteria* | *Pseudomonas* |
| OTU_8 | 2.08 | 3.88 | 29.76 | *Proteobacteria* | *Gammaproteobacteria* | Unclassified |
| OTU_4 | 1.77 | 3.28 | 33.05 | *Proteobacteria* | *Gammaproteobacteria* | *Lysobacter* |
| OTU_9 | 1.63 | 3.02 | 36.07 | *Actinobacteria* | *Actinobacteria* | *Williamsia* |
| OTU_6 | 1.61 | 2.99 | 39.06 | *Proteobacteria* | *Gammaproteobacteria* | Unclassified |
| OTU_10 | 1.36 | 2.53 | 41.59 | *Proteobacteria* | *Alphaproteobacteria* | *Parvibaculum* |
| OTU_2 | 1.22 | 2.27 | 43.86 | *Proteobacteria* | *Betaproteobacteria* | Unclassified |
| OTU_13 | 1.06 | 1.97 | 45.82 | *Bacteroidetes* | *Flavobacteriia* | Unclassified |
| OTU_15 | 1.03 | 1.92 | 47.74 | *Bacteroidetes* | *Bacteroidia* | *Mangrovibacterium* |
| OTU_11 | 1.02 | 1.89 | 49.63 | *Bacteroidetes* | Unclassified | Unclassified |
| OTU_25 | 0.89 | 1.66 | 51.29 | *Proteobacteria* | *Gammaproteobacteria* | *Povalibacter* |
| OTU_27 | 0.84 | 1.57 | 52.86 | *Proteobacteria* | *Alphaproteobacteria* | *Brevundimonas* |
| OTU_16 | 0.80 | 1.49 | 54.35 | *Proteobacteria* | *Gammaproteobacteria* | *Solimonas* |
| OTU_14 | 0.75 | 1.40 | 55.75 | *Bacteroidetes* | *Sphingobacteriia* | *Sediminibacterium* |
| OTU_22 | 0.73 | 1.35 | 57.11 | *Proteobacteria* | *Alphaproteobacteria* | *Sphingobium* |
| OTU_21 | 0.72 | 1.35 | 58.45 | *Proteobacteria* | *Deltaproteobacteria* | *Smithella* |
| OTU_18 | 0.71 | 1.33 | 59.78 | *Bacteroidetes* | *Bacteroidia* | *Paludibacter* |
| OTU_19 | 0.70 | 1.30 | 61.09 | Unclassified | Unclassified | Unclassified |
| OTU_26 | 0.66 | 1.23 | 62.32 | *Proteobacteria* | *Betaproteobacteria* | *Comamonas* |
| OTU_20 | 0.56 | 1.05 | 63.37 | *Bacteroidetes* | *Bacteroidia* | *Petrimonas* |
| OTU_12 | 0.55 | 1.02 | 64.39 | *Proteobacteria* | *Gammaproteobacteria* | *Pseudoxanthomonas* |
| OTU_23 | 0.54 | 1.00 | 65.39 | *Chloroflexi* | *Anaerolineae* | Unclassified |
| OTU_28 | 0.52 | 0.97 | 66.36 | *Bacteroidetes* | *Cytophagia* | *Dyadobacter* |
| OTU_24 | 0.51 | 0.94 | 67.30 | *Bacteroidetes* | *Bacteroidia* | *Mariniphaga* |
| OTU_82 | 0.49 | 0.90 | 68.21 | *Proteobacteria* | *Betaproteobacteria* | *Simplicispira* |
| OTU_53 | 0.46 | 0.85 | 69.06 | *Proteobacteria* | *Gammaproteobacteria* | Unclassified |
| OTU_133 | 0.46 | 0.85 | 69.91 | *Bacteroidetes* | *Bacteroidia* | *Petrimonas* |

**Supplemental Table S4.** Average dissimilarity values obtained after pairwise SIMPER (similarity percentage) analysis of OTU-based fungal community structures in the contaminated soil at initial conditions (t0) and after 15 weeks without fertilization at 10 (UNF10) and 20 °C (UNF20) and with NPK fertilization at 10 (NPK10) and 20°C (NPK20).

| | t0 | UNF10 | UNF20 | NPK10 | NPK20 |
|---|---|---|---|---|---|
| t0 | | | | | |
| UNF10 | 69.88 | | | | |
| UNF20 | 84.40 | 50.21 | | | |
| NPK10 | 60.96 | 76.30 | 95.08 | | |
| NPK20 | 74.90 | 84.55 | 93.79 | 89.84 | |

**Supplemental Table S5**. Relative abundances (%) of the fungal phyla and subphylum as well as the most abundant fungal classes found in the contaminated soil at initial conditions (t0) and after 15 weeks without fertilization at 10 (UNF10) and 20 °C (UNF20) and with NPK fertilization at 10 (NPK10) and 20°C (NPK20). For each taxonomic group, mean values followed by different letters are significantly different ($p \leq 0.05$) according to Tukey's HSD test. Results of correlation anaylses (Pearson method) between contents of total petroleum hydrocarbons (TPH) and relative abundances of the different taxonomic groups considering the five treatments are also given; R values in bold denote statistical significance ($p \leq 0.05$).

| **Taxa** | | **Experimental treatments** | | | | | **Correlations with TPH contents** |
|---|---|---|---|---|---|---|---|
| | | **t0** | **UNF10** | **UNF20** | **NPK10** | **NPK20** | **R (*p-value*)** |
| *Ascomycota* (phylum) | | 23.34 **b** | 17.04 **ab** | 3.09 **a** | 7.31 **ab** | 91.60 **c** | -0.4398 (0.1009) |
| *Ascomycota* classes | *Dothideomycetes* | 0.04 **a** | 5.50 **a** | 0.20 **a** | 0.03 **a** | 0.06 **a** | -0.0802 (0.7763) |
| | *Eurotiomycetes* | 1.58 **a** | 0.95 **a** | 0.01 **a** | 1.21 **a** | 0.01 **a** | 0.4505 (0.092) |
| | *Leotiomycetes* | 0.01 **a** | 0.96 **a** | 0.22 **a** | 0.00 **a** | 0.01 **a** | -0.0528 (0.8516) |
| | *Sordariomycetes* | 2.71 **a** | 0.05 **a** | 0.38 **a** | 0.03 **a** | 7.00 **a** | -0.1541 (0.5834) |
| | Incertae sedis 14 | 0.00 **a** | 0.54 **a** | 0.00 **a** | 0.00 **a** | 0.01 **a** | 0.0169 (0.9564) |
| *Basidiomycota* (phylum) | | 6.98 **a** | 38.67 **b** | 59.93 **c** | 1.43 **a** | 2.27 **a** | -0.1614 (0.5655) |
| *Basidiomycta* classes | *Agaricomycetes* | 1.18 **a** | 12.86 **b** | 4.05 **a** | 0.09 **a** | 0.11 **a** | -0.0561 (0.8427) |
| | *Tremellomycetes* | 0.01 **a** | 0.01 **a** | 1.36 **a** | 0.01 **a** | 0.01 **a** | -0.1019 (0.718) |
| | Incertae sedis 4 | 0.02 **a** | 0.03 **a** | 0.00 **a** | 0.78 **a** | 1.19 **a** | -0.3951 (0.145) |
| *Mucoromycotina* (subphylum) | | 0.73 **a** | 0.01 **a** | 0.00 **a** | 1.57 **a** | 0.01 **a** | 0.2285 (0.4128) |
| *Mucoromycotina* classes | Incertae sedis 10 | 0.73 **a** | 0.01 **a** | 0.00 **a** | 1.57 **a** | 0.01 **a** | 0.2281 (0.4136) |
| Unclassified (phylum level) | | 68.96 **c** | 44.28 **b** | 36.98 **b** | 89.69 **d** | 6.12 **a** | **0.6228** (0.0131) |
| | Unclassified (class level) | 93.72 **ab** | 79.09 **a** | 93.77 **ab** | 96.28 **ab** | 91.59 **b** | 0.1076 (0.7027) |

**Supplemental Table S6**. Taxonomic classification of the top 20 OTUs contributing to dissimilarities (similarity percentage analysis) in fungal community structures among the five treatments (t0, UNF10, UNF20, NPK10, NPK20) using a 50% confidence threshold (ribosomal database project taxonomic classifier). OTUs were ordered in decreasing order of dissimilarity contribution.

| OTU number | Average dissimilarity | Dissimilarity contribution (%) | Cumulative diss. contribution (%) | Domain | Phylum | Class | Genus |
|---|---|---|---|---|---|---|---|
| OTU_4 | 18.03 | 23.12 | 23.12 | Fungi | *Ascomycota* | Unclassified | Unclassified |
| OTU_1 | 13.23 | 16.97 | 40.08 | Fungi | *Basidiomycota* | Unclassified | Unclassified |
| OTU_3 | 11.54 | 14.79 | 54.88 | Fungi | Unclassified | Unclassified | Unclassified |
| OTU_2 | 9.84 | 12.62 | 67.49 | Fungi | Unclassified | Unclassified | Unclassified |
| OTU_5 | 5.36 | 6.87 | 74.36 | Fungi | Unclassified | Unclassified | Unclassified |
| OTU_6 | 4.03 | 5.17 | 79.53 | Fungi | Unclassified | Unclassified | Unclassified |
| OTU_7 | 2.18 | 2.79 | 82.32 | Fungi | Unclassified | Unclassified | Unclassified |
| OTU_8 | 1.10 | 1.40 | 83.72 | Fungi | *Ascomycota* | *Sordariomycetes* | Sordariomycetes_unidentified_1 |
| OTU_9 | 0.86 | 1.10 | 84.82 | Fungi | *Basidiomycota* | *Agaricomycetes* | Unclassified |
| OTU_11 | 0.73 | 0.94 | 85.76 | Fungi | *Ascomycota* | *Dothideomycetes* | Pleosporales_unidentified_1 |
| OTU_10 | 0.72 | 0.93 | 86.69 | Fungi | Unclassified | Unclassified | Unclassified |
| OTU_12 | 0.62 | 0.79 | 87.48 | Fungi | Unclassified | Unclassified | Unclassified |
| OTU_38 | 0.49 | 0.63 | 88.11 | Fungi | Unclassified | Unclassified | Unclassified |
| OTU_14 | 0.48 | 0.61 | 88.72 | Fungi | *Basidiomycota* | *Agaricomycetes* | *Cylindrobasidium* |
| OTU_27 | 0.42 | 0.54 | 89.26 | Fungi | Unclassified | Unclassified | Unclassified |
| OTU_19 | 0.40 | 0.52 | 89.78 | Fungi | Unclassified | Unclassified | Unclassified |
| OTU_17 | 0.36 | 0.47 | 90.24 | Fungi | Unclassified | Unclassified | Unclassified |
| OTU_15 | 0.35 | 0.46 | 90.70 | Fungi | *Basidiomycota* | *Agaricomycetes* | *Fomitopsis* |
| OTU_22 | 0.32 | 0.41 | 91.11 | Fungi | *Ascomycota* | *Saccharomycetes* | *Candida* |
| OTU_25 | 0.32 | 0.41 | 91.52 | Fungi | *Ascomycota* | *Eurotiomycetes* | *Penicillium* |
